# Synthetic long oligonucleotides to generate artificial templates for use as positive controls in molecular assays: drug resistance mutations in influenza virus as an example

**DOI:** 10.1186/1743-422X-8-405

**Published:** 2011-08-16

**Authors:** Bin Wang, Megan C Steain, Dominic E Dwyer, Anthony L Cunningham, Nitin K Saksena

**Affiliations:** 1 Retroviral Genetics Laboratory, Centre for Virus Research, Westmead Millennium Institute, Westmead Hospital, The University of Sydney, Westmead, New South Wales 2145, Australia; 2 Department of Infectious Diseases and Immunology, University of Sydney, Blackburn Building, 2006 NSW Australia; 3 Centre for Infectious Diseases and Microbiology (CIDM), Institute of Clinical Pathology and Medical Research (ICPMR), Westmead, New South Wales 2145, Australia

## Abstract

**Background:**

Positive controls are an integral component of any sensitive molecular diagnostic tool, but this can be affected, if several mutations are being screened in a scenario of a pandemic or newly emerging disease where it can be difficult to acquire all the necessary positive controls from the host. This work describes the development of a synthetic oligo-cassette for positive controls for accurate and highly sensitive diagnosis of several mutations relevant to influenza virus drug resistance.

**Results:**

Using influenza antiviral drug resistance mutations as an example by employing the utility of synthetic paired long oligonucleotides containing complementary sequences at their 3' ends and utilizing the formation of oligonucleotide dimers and DNA polymerization, we generated ~170bp dsDNA containing several known specific neuraminidase inhibitor (NAI) resistance mutations. These templates were further cloned and successfully applied as positive controls in downstream assays.

**Conclusion:**

This approach significantly improved the development of diagnosis of resistance mutations in terms of time, accuracy, efficiency and sensitivity, which are paramount to monitoring the emergence and spread of antiviral drug resistant influenza strains. Thus, this may have a significantly broader application in molecular diagnostics along with its application in rapid molecular testing of all relevant mutations in an event of pandemic.

## Background

Recently, the occurrence in humans of infection with virulent avian influenza A H5N1 and the emergence of swine origin pandemic influenza A H1N1 2009 strain have sparked fear of an ongoing pandemic with novel genetic characters [1-3]. While vaccines remain the most effective public health strategy for prevention [4,5], antiviral drugs such as neuraminidase inhibitors (NAIs), oseltamivir and zanamivir, could play an important role in the response to the early phases of a pandemic, if available in sufficient quantities. However, like other antiviral agents, the emergence of influenza viruses with reduced susceptibility to the NAI is inevitable during treatment [1]. To date, strains with altered susceptibility to NAI have been recovered from approximately 1% of immunocompetent adult patients [6] and up to 18% of pediatric patients [7]. In addition, oseltamivir-resistant influenza A H5N1 and pandemic H1N1 2009 viruses with the H274Y mutation have been reported from patients during oseltamivir treatment [8,9].

Significant advances in molecular biology and human genomic research has paved the way for a host of new genetic diagnostic tests, including gene sequencing, detection, identification and genotyping of organisms using real time polymerase chain reaction (PCR) or other amplification techniques such as multiplex PCR, reverse line blot hybridization (RLB) [10], Ligase chain reaction (LCR), Rolling Circle Amplification (RCA) [9,11], microarray [12-14]. Sequencing often serves as a 'gold standard' for the detection of single nucleotide polymorphisms, drug resistance mutations or virus/bacterial typing [15,16]. However, as sequencing is cumbersome, expensive and less likely to detect low prevalence mutations (mutations consisting less than 30% of total populations) [17,18], newer alternative techniques such as real time PCR and RCA are being employed [9,15]. To establish molecular assays, positive controls are a prerequisite to ascertain specificity and sensitivity [19,20]. However, in many cases it is difficult and cumbersome to acquire appropriate positive controls. It is thus, important to mention that although the commercial oligonucleotide synthesis can generate long oligonucleotides (≥150 mers) and can serve as suitable controls, there are problems as the oligonucleotide length exceeds 100 nucleobases, the yield of desired products often becomes limited by side reactions and even modest inefficiencies within the stepwise chemical reactions can have large effects on the final yield [21].

Further, in the event of an influenza pandemic, drug resistant strains and their transmission could be clinically highly significant [1], meaning that sensitive and specific techniques are required for their early and clinically relevant detection. However, due to the low frequency of naturally occurring resistance mutations in influenza infected patients receiving NA inhibitor treatment, the highly pathogenetic nature of influenza A H5N1 strains, and the technical complexity and time consuming nature of generating of NA resistant strains *in vitro*, collection of all known resistance mutations as positive controls is challenging. Therefore, our approach utilizing the formation of oligonucleotide dimers between two commercially synthesised long single stranded DNA templates (~95 bases each), to generate ~170 bp double-stranded artificial DNA templates, containing resistance mutations is not only innovative, but a more molecularly feasible and durable. In this way, it offers significant advantages in synthesizing even longer double stranded DNA templates, which can be used as positive control templates in molecular diagnostics. In the present study, we have used these double stranded artificial DNA templates with all known genetic mutations associated with influenza A drug resistance as a positive control in the development of a ligase chain reaction (LCR) for detecting NA inhibitor drug resistance mutations in patient samples.

## Results

### Generation of ds DNA template from synthetic oligos

**Figure 1:**
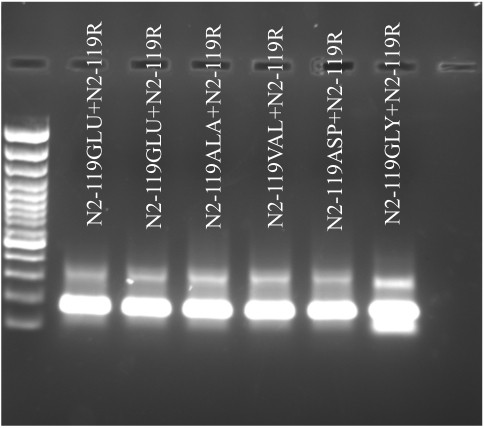
**Generation of double stranded DNA templates using paired synthetic long oligos**. After 5 cycles of PCR, the products were run on 2% agarose gel. The formation of ~170 bp of product can be clearly visualized.

After 5 PCR reaction cycles, the products from paired synthetic long oligos were run on 2% agarose gel, stained with ethidium bromide and viewed under UV light. The dsDNA products were clearly visualized at the size ~170 bp (Figure 1), demonstrating a successful production of long ds DNA templates from synthetic oligos.

### Cloning of DNA products and sequencing of positive clones

The PCR products from each paired oligo were cloned and 5 positive clones were sequenced to validate the presence of desired mutations. Each sequence was aligned carefully and compared with the artificial template and reference strains. As expected, most sequences were identical to the artificial template design carrying resistance mutations (Table 1).

**Table 1:** List of artificial templates containing wild type and NA-resistance mutations.

Oligo name	Oligo Sequence	Location in reference strains	Description
N1 274H 95nt	5'TCGTACAAAATCTTCAAGATCGAAAAGGGAAAGGTTACTAAATCAATAGAGTTGAATGCACCCAATTTTCATTATGAGGAATGTTCCTGTTACCC 3'	H1N1 (CY12306.1, 763-857)	Wild Type
N1 274Y 95nt	5'TCGTACAAAATCTTCAAGATCGAAAAGGGAAAGGTTACTAAATCAATAGAGTTGAATGCACCCAATTTTTATTATGAGGAATGTTCCTGTTACCC 3'	H1N1 (CY12306.1, 763-857)	Resistance mutation
N1 274R 96nt	5'TTGATTAAAAGACACCCAAGGTCGATTTGAACCATGCCAGTTGTCCCTGCATACACACATCACTGTGCCAGTGTCTGGGTAACAGGAACATTCCTC3'	H1N1 (CY12306.1, 933-837)	Antisense oligo
H5N1 274H 95nt	5'TCACATAAGATCTTCAAAATGGAAAAAGGGAAAGTGGTTAAATCAGTCGAATTGGATGCTCCTAATTATCACTATGAGGAATGCTCCTGTTATCC3'	H5N1 (DQ493076, 694-788)	Wild Type
H5N1 274Y 95nt	5'TCACATAAGATCTTCAAAATGGAAAAAGGGAAAGTGGTTAAATCAGTCGAATTGGATGCTCCTAATTATTACTATGAGGAATGCTCCTGTTATCC3'	H5N1 (DQ250165, 694-788)	Resistance mutation
H5N1 274R 96nt	5'TTGATTGAAAGATACCCATGGCCGATTTGAGCCATGCCAATTATCCCTGCACACACATGTGATTTCGCCGGCATCAGGATAACAGGAGCATTCCTC3'	H5N1 (DQ250165, 864-768)	Antisense oligo
N2-119GLU 90nt	5'TCTAAGGACAATTCGATTCGGCTTTCCGCTGGTGGGGACATCTGGGTGACAAGAGARCCTTATGTGTCATGCGATCCTGACAAGTGTTATC 3'	H3N2 (CY016653,305-395)	Wild Type
N2-119Val 90nt	5'TCTAAGGACAATTCGATTCGGCTTTCCGCTGGTGGGGACATCTGGGTGACAAGAGTNCCTTATGTGTCATGCGATCCTGACAAGTGTTATC3'	H3N2 (CY016653,305-395)	Resistance mutation
N2-119GLY 90nt	5'TCTAAGGACAATTCGATTCGGCTTTCCGCTGGTGGGGACATCTGGGTGACAAGAGGNCCTTATGTGTCATGCGATCCTGACAAGTGTTATC3'	H3N2 (CY016653, 305-395)	Polymorphisms
N2-119ALA 90nt	5'TCTAAGGACAATTCGATTCGGCTTTCCGCTGGTGGGGACATCTGGGTGACAAGAGCNCCTTATGTGTCATGCGATCCTGACAAGTGTTATC3'	H3N2 (CY016653,305-395)	Polymorphisms
N2-119ASP 90nt	5'TCTAAGGACAATTCGATTCGGCTTTCCGCTGGTGGGGACATCTGGGTGACAAGAGAYCCTTATGTGTCATGCGATCCTGACAAGTGTTATC3'	H3N2 (CY016653, 305-395)	Polymorphisms
N2-119R 98nt	5'CCGATAAGGGGTCCTATCATGTACTGTGTCATTTGAATGCCCGTTGTTTAGTGTTGTTCCCTGTCCAAGGGCAAATTGATAACACTTGTCAGGATCGC3'	H3N2 (CY016653, 472-375)	Antisense oligo
N2-292ARG 89nt	CAGCATGTCGAGGAGTGCTCCTGTTATCCTCGATATCCTGGTGTCAGATGTGTCTGCAGRGACAACTGGAAAGGCTCCAATAGGCCCAT3'	H3N2 (CY016653, 821-909	Wild Type
N2-292LYS 89nt	5'CAGCATGTCGAGGAGTGCTCCTGTTATCCTCGATATCCTGGTGTCAGATGTGTCTGCAARGACAACTGGAAAGGCTCCAATAGGCCCAT3'	H3N2 (CY016653, 821-909	Resistance mutation
N2-292R 99nt	5'TTTCTGGGTGTGTCTCCAACAAGTCCTGAGCACACATAACTGGAAACAATGCTATAATCCTTTACATTTATATCTACGATGGGCCTATTGGAGCCTTTC3'	H3N2 (CY016653, 987-889	Antisense oligo

1: Synthetic templates containing the wild type, naturally occurring polymorphisms and resistance mutations. These genome locations were indicated in the table according to reference strains.

2: Antisense oligo (R) with complementary sequences at 3' end were also synthesised and the complementary sequence were shown as underline.

### Ligase Chain Reaction in the detection of resistance mutation

**Figure 2:**
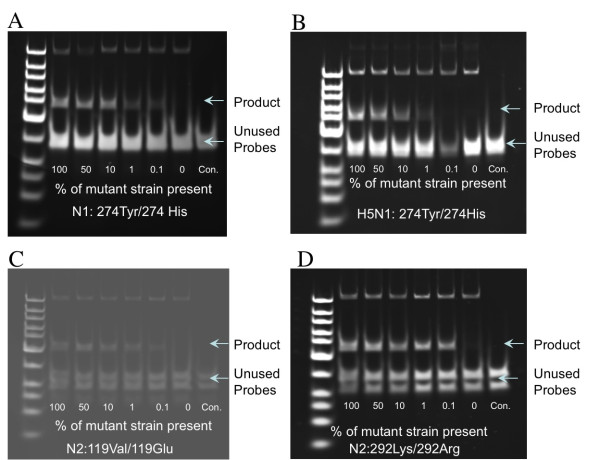
**Ligase chain reaction testing standard containing resistance template at various levels**. A: N1: 274Tyr (resistance)/His (wild type); B: H5N1: 274Tyr (resistance)/His (wild type). C: N2:119Val(resistance)/Glu(wild type), D: N2:292Lys(resistance)/Arg(wild type). After 10 cycle of ligation reaction, the generation of positive product can be detected even in the presence of low-level of resistance mutation (~1%).

Standard liner template containing resistance mutations (N1: His274Tyr, H5N1 His274Tyr, N2 Glu119Val, and N2 Ag292Lys) at various levels were prepared by mixing wild-type template with template containing resistance mutations. Each standard template was targeted by four independent probes specifically recognizing each of the resistance mutation. After 10 cycles of ligation reaction, even the presence of low-level of resistance mutation (~1%) could be detected, as evidence by the formation of specific dsDNA molecules of about 80-90 bp (Figure 2).

## Discussion and Conclusions

Although sequencing serves as a 'gold standard' for the detection of single nucleotide polymorphisms associated with drug resistance mutations or organism typing methods [15,16], alternative techniques are being employed [9,15,22,23]. One impediment to these techniques is the lack of availability of control templates carrying rare mutant variants/alleles or all relevant mutations, which can serve as positive controls in molecular or multiplex PCR assays simultaneously. In influenza studies, for example, several single nucleotide mutations are known to be associated with NA inhibitor resistance [24,25]. Many of these have been derived from different *in vitro *cultures, and currently naturally occurring NAI resistance appears to be uncommon in some strains. However, in the event of an influenza pandemic, the use of NAIs would dramatically increase and thus provide greater opportunities for resistance to emerge and spread. Therefore, rapid screening of these mutations, which would be of great benefit for patient treatment and the control of disease spread, should be available in a format that can facilitate simultaneous, rapid and bulk detection of drug resistance mutations. We were only able to source wild-type clinical isolates of influenza A H3N2; known resistant clinical strains were unavailable. Given that highly pathogenic avian influenza A H5N1 can only be handled in PC4 facilities, we had only limited access to influenza A H5N1 RNA. This was the main impediment to molecular assay development for the detection of drug resistance mutations. Thus, we endeavored to create artificial templates containing all previously identified single nucleotide mutations known for drug resistance within the neuraminidase gene of influenza virus. To address this issue, we produced long synthetic oligos containing known influenza A resistance mutations, and showed them to be effective as controls in LCR. Further, they can also serve as templates in real-time PCR and RCA [23,9].

The use of fully synthetic oligonucleotides as positive control material has been described recently [16]. However, there were limitations as to the length of the oligonucleotide, which was generally unable to reach more than 130 bases. In many situations, for instance in the case of drug resistance mutations, nucleotide substitutions are often scattered within the target genome and thus require larger fragments as standard templates. Here, we took a novel approach to creating long artificial templates by taking advantage of primer-dimers, which are usually considered a common laboratory problem. The binding of two long artificially synthesized oligos generated a substrate for PCR and was used to create long double-stranded DNA molecules of about 170 bp. In most of the current diagnostic studies (especially based on real time PCR and RCA), 150-240 bp would be sufficient to serve a template controls. However, it is possible to use multiple long oligos to synthesize even longer double-stranded DNA and up to the full length of some important genes, if need be [26,27], which is a significant advantage over any similar technologies existing currently.

The most significant advantage of using synthetic oligonucleotides is flexibility. Our approach has proven that the use of laboratory safe artificial templates can be a relatively cost effective, simple and efficient alternative to difficult to acquire material related to an infection or bioterrorism agent. The production of standard templates via the formation of oligo dimers and PCR using synthetic oligos does not require the use of viral or bacterial strains and oligos of any desired sequence can be made commercially. This approach may have the potential to produce a false-positive product due to contamination and therefore caution may be necessary. However, this problem can be solved by the introduction of exclusive restriction endonuclease digestion sites in the artificial template, which would allow for rapid confirmation of a false-positive result. Certain restriction endonuclease (*Sma*I and *BamH*I) can be used directly in the PCR buffer (without buffer exchange) and thus the PCR product from the control can be easily and rapidly distinguished from testing samples. In addition, the standard artificial template could be designed to be slightly smaller or larger in size, which would allow for direct visual identification of contamination with the synthetic control.

Overall, this technology may have wide ranging applications and may revolutionize molecular diagnostics and the use of multiplexing assays.

## Methods

### Reference strains

The reference strains of influenza A H1N1, H5N1 and H3N2 used in this study had Genebank accession numbers: CY12306.1, DQ493076, CY016653, respectively.

### Design and synthesis of long oligos

Long single-stranded oligonucleotides of 89-98 bases were designed, each containing wild-type template or drug resistance mutations (Table 1). After synthesis, each oligonucleotide was PAGE purified (Sigma-Aldrich, Sydney, Australia). Paired long oligos were designed carrying >20bp complementary sequences at their 3' ends.

### Generation of double-stranded DNA using long oligos and Taq polymerase

20 pmol of each paired oligo was incubated with 2 U of *Taq *DNA polymerase (Promega, Madison, USA), 1X reaction buffer, 2.5 mM MgCl_2_, and 0.4 mM of dNTPs. The reaction mix was incubated at 94°C for 30 seconds followed by 55°C for 30 seconds and 72°C for 30 seconds for 5 cycles. After the reaction, the end product was run on a 2% agarose gel and visualized under UV light with ethidium bromide staining.

**Figure 3:**
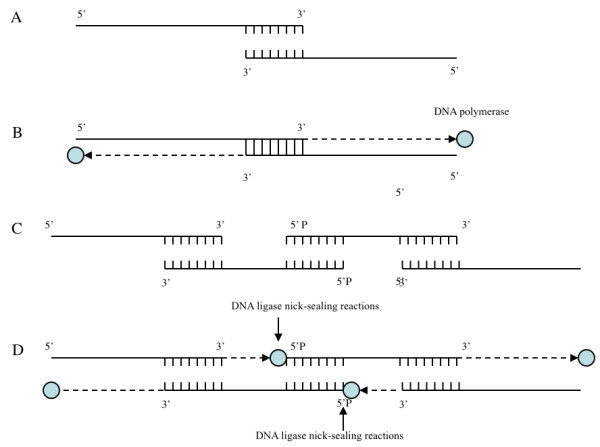
**Schematic representation of generating large DNA molecule using synthetic oligonucleotide templates**. (A) The formation of a dimer from two synthetic oligonucleotide templates. (B) In the presence of dNTP and DNA polymerase, each oligonucleotide will extend its 3' end using other oligo as a template and form double-stranded DNA molecules. (C) The theory can be further extended using multiple oligos containing complementary sequences at the 5'-phospahte ends. (D) Upon DNA polymerase reaching the 5' end an oligo where a second down stream oligo is annealed, the adjacent 3'-hydroxy ends and 5'-phosphate (P) can be sealed by DNA ligase via nick-sealing reactions. This process in theory can be used to generate a very large, double-stranded DNA fragment.

During this process, the complementary sequences located at 3' end of the paired oligo would form a dimer and in the presence of DNA polymerase and dNTP, each oligo would extend its 3' end using the second oligo as a template (Figure 3).

### Cloning of the double-stranded DNA

To ensure the purity of the standard template, the products were ligated into the pGEM-T Easy Vector System II (Promega, Madison, USA) and transformed into competent *Escherichia coli *JM109 cells, according to the manufacturer's protocol. Screening for the gene insert was performed by quick lysis at 95°C of the *E. coli *cells for 5 minutes, followed by PCR using 5 μl of the cell lysate as previously described [28].

### Sequencing of cloned dsDNA and sequence analysis

The plasmid DNA from positive clones was extracted using the QIAprep Spin Miniprep Kit according to the manufacturer's protocol (Qiagen, Melbourne, Australia). The inserts were sequenced using Applied Biosystems BigDye terminator chemistry version 3.1 (Foster City, CA, USA), on an ABI Prism 373 DNA sequencer. Sequences were identified using the FastA program group accessed through Biomanager (http://biomanager.info/).

### Ligase Chain Reaction (LCR) in the detection of templates containing resistance mutation

Liner templates containing resistance and wild type were generated by PCR amplification of plasmid DNA, followed by purification using Millipore PCR purification plate (Millipore, Billerica, MA, USA). The linear PCR products were quantitated using spectrophotometer and dsDNA DNA copy number were estimated by DNA calculator (http://www.uri.edu/research/gsc/resources/cndna.html). 5 × 10^11 ^copies of standard templates were used for testing the specificity of the LCR system. LCR probes targeting resistance template were designed (Table 2). Each template was targeted by four LCR probes about 35-45nt with 2 of the probes having 5' end phosphorylation. Ligation of LCR probes to standard templates was carried out by mixing the standard template with 1pmol of each LCR probes, 2U of pfu DNA ligase (Stratagene, Integrated Sciences, Cedar Creek, TX, USA) in 20 mM Tris-HCl (pH 7.5), 20 mM KCl, 10 mM MgCl_2_, 0.1% Igepal, 0.01 mM rATP, 1 mM DTT with total reaction volume of 25 μl. Multiple cycle ligation was conducted to validate the specificity of the probe in recognizing its corresponding template. The reaction condition include one cycle of 5 min at 94°C to denature the dsDNA followed 10 cycles of by 94°C 30 s and 4 min ligation at 65°C. The final product were run on 10% TBE gel (Invitrogen, Mount Waverley VIC Australia) and visualized under UV light with ethidium bromide staining.

**Table 2:** LCR probes for resistance mutations detection

Mutation type	Probe sequences
N2-119VAL	5' AAGGGGACATCTGGGTGACAAGAGT 3'
	5' ^a^P-NCCTTATGTGTCATGCGATCCTGACAA 3'
	5' ACCTTGTCAGGATCGCATGACACATAAGGNA 3'
	5' P-CTCTTGTCACCCAGATGTCCCCAC 3'
N2-292LYS	5' ATGATATCCTGGTGTCAGATGTGTCTGCAA 3'
	5' P-RGACAACTGGAAAGGCTCCAATAGGC 3'
	5' CCCCTATTGGAGCCTTTCCAGTTGTCYT 3'
	5' P-TGCAGACACATCTGACACCAGGATATCGAG 3'
N1-274Y	5' TGTTACTAAATCAATAGAGTTGAATGCACCCAATTTTT 3'
	5' P-ATTATGAGGAATGTTCCTGTTACCCACACAC 3'
	5' TTGTGTCTGGGTAACAGGAACATTCCTCATAATA 3'
	5' P-AAAATTGGGTGCATTCAACTCTATTGATTTAGTAACC 3'
H5N1-274Y	5' TCGGTTAAATCAGTCGAATTGGATGCTCCTAATTATT 3'
	5' P-ACTATGAGGAATGCTCCTGTTATCCTGATG 3'
	5' TTGCATCAGGATAACAGGAGCATTCCTCATAGTA 3'
	5' P-ATAATTAGGAGCATCCAATTCG 3'

a. P- indicates 5' phosphorylation

## List of abbreviations

NAI: Neuraminidase inhibitor; PCR: polymerase chain reaction; RLB:reverse line blot hybridization; LCR: Ligase chain reaction; RCA: Rolling Circle Amplification.

## Competing interests

The authors declare that they have no competing interests.

## Authors' contributions

BW conceived, designed and conducted the study along with sequence search, data analyses and manuscript writing. MS assisted with the assay optimization, cloning, DNA sequencing. DD, AC and NS provided intellectual input and manuscript writing. All authors have read and approved the manuscript.
